# Insulin-like growth factor-1 and insulin-like growth factor binding protein 3 and risk of postoperative cognitive dysfunction

**DOI:** 10.1186/s40064-015-1586-2

**Published:** 2015-12-18

**Authors:** Jue Jiang, Zhifeng Chen, Bing Liang, Jia Yan, Ying Zhang, Hong Jiang

**Affiliations:** Department of Anesthesiology, Shanghai Ninth People’s Hospital, Shanghai Jiao Tong University School of Medicine, Zhizaoju Road 639, Shanghai, 200011 China

**Keywords:** Postoperative cognition dysfunction, Insulin-like growth factor-1, Insulin-like growth factor binding protein3, General anesthesia

## Abstract

Insulin-like growth factor (IGF)-1 is implicated in learning and memory. Experimental studies have suggested that the IGF-1 system is beneficial in cognition, especially in Alzheimer’s disease (AD), by opposing Aβ amyloid processing and hyperphosphorylated tau toxicity. Low IGF-I and insulin-like growth factor binding protein (IGFBP)-3 serum levels are significantly associated with AD. To assess the relationship between circulating IGF-I and IGFBP3 levels and change of postoperative cognition. The study was performed in patients scheduled for elective head and neck carcinoma surgery under general anesthesia. On the day before the operation and postoperative days 1, 3 and 7, mini-mental state examination (MMSE) was performed by the same doctor, and blood samples were collected at 08:00 h after overnight fasting. The circulating levels of IGF-1 and IGFBP3 were measured by enzyme-linked immunosorbent assay. One hundred and two patients completed all four MMSE tests and forty-four of them completed all the four blood samples collection. Postoperative circulating IGF-1 level, ratio of IGF-1/IGFBP3 and MMSE score significantly decreased, whereas IGFBP3 level significantly increased compared with preoperative values in total patients. The change trends of circulating IGF-1 level and MMSE score were similar. Preoperative circulating IGF-1 level, ratio and MMSE score were significantly lower in POCD group compared to non-POCD group. There was no significant difference in preoperative level of circulating IGFBP3 between the two groups. Preoperative circulating IGF-1 level was negatively correlated with age and positively with MMSE. Logistic regression analysis revealed that lower preoperative IGF-1 level and elderly patients increased the odds of POCD. Down-regulation of circulating IGF-1 level may be involved in the mechanism of postoperative cognitive dysfunction. Older patients had lower circulating IGF-1 levels and were more susceptible to POCD.

## Background

Since Savageau first described an association between postoperative cognitive dysfunction (POCD), surgery and anesthesia exposure in 1982 (Hartmann et al. 2007), many studies have documented the onset of POCD, which manifests as a decline in brain function, typically resolving within 12 months. Although POCD may only last for a short period (days or weeks) in most patients after cardiac and non-cardiac surgery, POCD in some patients can last for several months or longer, and even increase mortality (Nelson et al. 2012). It is conceivable that general anesthesia may contribute to POCD. Rats exposed to volatile anesthetics develop cognitive impairment (Duyckaerts et al. 2009), and β-amyloid peptide (Aβ) production is increased in mouse brains after volatile anesthetic exposure (Carro and Torres-Aleman 2006). Aβ oligomerization in vitro can be induced by volatile anesthetics (Saenger et al. 2011). It has been proposed that Aβ overproduction, oligomerization and accumulation in the brain contribute to the development of Alzheimer’s disease (AD) (Cohen et al. 2009), the most common form of dementia in elderly patients.

It was reported that insulin-like peptide signaling (ILPs) (includes IGF-1 and IGF-2) correlated with sporadic AD (Piriz et al. 2011). Evidence gathered from human studies shows a positive correlation between insulin-like growth factor (IGF)-I levels and mental ability (Lan et al. 2012), while cognitive impairment has been found in human patients affected by growth hormone/IGF-I deficiency (Hanning 2005). Administration of sevoflurane might temporally affect the ability of cognitive function in rats, through suppressing IGF-1 mRNA expression in the hippocampus (Kuningas et al. 2008). Although IGF-1 and its receptor and binding proteins are locally produced in the brain (Alvarez et al. 2007), IGF-1 is actively transported across the blood–brain barrier, and therefore changes in circulating IGF-1 can lead to changes in IGF-1 input to the brain (Creyghton et al. 2004). The bioavailability and bioactivity of IGF-1 is regulated by six IGFBPs (IGFBP1-6) and several IGFBP proteases (Carro and Torres-Aleman 2004). Quantitatively the most important binding protein in the circulation is IGFBP3 which binds >80 % of the circulating IGF-1 (Culley et al. 2003). Thus, measurement of circulating IGFBP3 levels, in addition to IGF-1 levels, allows the amount of bioavailable IGF-1 to be determined (Alvarez et al. 2006). In contrast to IGFBP1 to IGFBP6, which bind to the IGFs (Firth and Baxter 2002), IGFBP7 is a critical regulator of memory consolidation that can attenuate the function of ILPs (Agbemenyah et al. 2013) and can directly bind to the IGF-1R and thereby inhibit its activity (Evdokimova et al. 2012). The relationship between circulating IGFBP7 level and POCD had been discussed in previous work (Jiang et al. 2015).

Hence, the present study was designed to investigate the perioperative changes of circulating IGF-1 (total IGF-1), ratio of IGF-1/IGFBP3 (bioavailable IGF-1) and IGFBP3 levels and the risk of POCD.

## Methods

### Patient population

The study was performed in patients scheduled for elective head and neck carcinoma surgery under general anesthesia. The data can be seen in our previous work (Jiang et al. 2015). All patients underwent a standardized clinical evaluation that included medical history and cognitive function assessment (mini-mental state examination; MMSE) (Rosario 2010).

### MMSE test

MMSE is a 30-point scale that measures global cognitive function, with higher scores indicating better function, with scores <24 suggestive of cognitive impairment (Folstein et al. 1975). Patients with MMSE ≤23 or diagnosed with depression or delirium before operation, operation time <8 h were excluded. For statistical analysis, according to previous report (Linstedt et al. 2002), a decline of more than 10 % or 2 points in MMSE test was regarded as POCD. Patients were classified as having or not POCD according to this definition and were compared in terms of age, gender, MMSE scores, circulating IGF-1 and IGFBP3 levels, and et al.

### Anesthesia and surgery

The details of procedure of anesthesia and surgery can be seen in our previous work (Jiang et al. 2015).

### Assays

Reference to previous work (Jiang et al. 2015), On the day before the operation and postoperative days1, 3 and 7, MMSE was performed, circulating IGF-1 and IGFBP3 levels were measured and the ratio of IGF-1/IGFBP3 was calculated (recorded as MMSE^1^, MMSE^2^, MMSE^3^ and MMSE^4^, IGF-1^1^, IGF-1^2^, IGF-1^3^, IGF-1^4^, IGFBP3^1^, IGFBP3^2^, IGFBP3^3^ andIGFBP3^4^, and ratio^1^, ratio^2^, ratio^3^, ratio^4^ respectively). According to the manufacturer’s data sheets, assay range for the IGF-1 was 10–200 and 5–100 μg/L for the IGFBP3 assay.

### Statistical analysis

All statistical analyses were performed using Stata12.0 and P ≤ 0.05 was considered to be statistically significant. Normal data are presented as mean ± SD. The paired or unpaired *t* test was used to compare mean values of normally distributed data. Differences in categorical data (expressed as percentages) were assessed using the χ^2^ test. Logistic regression analysis was used to investigate factors contributing to the risk of POCD. The model of logistic regression includes age, gender, height, weight, body mass index (BMI), education level, MMSE, IGF-1, IGFBP3 and ratio. Correlation analysis was used to illustrate the relationship of different parameters.

## Results

**Fig. 1:**
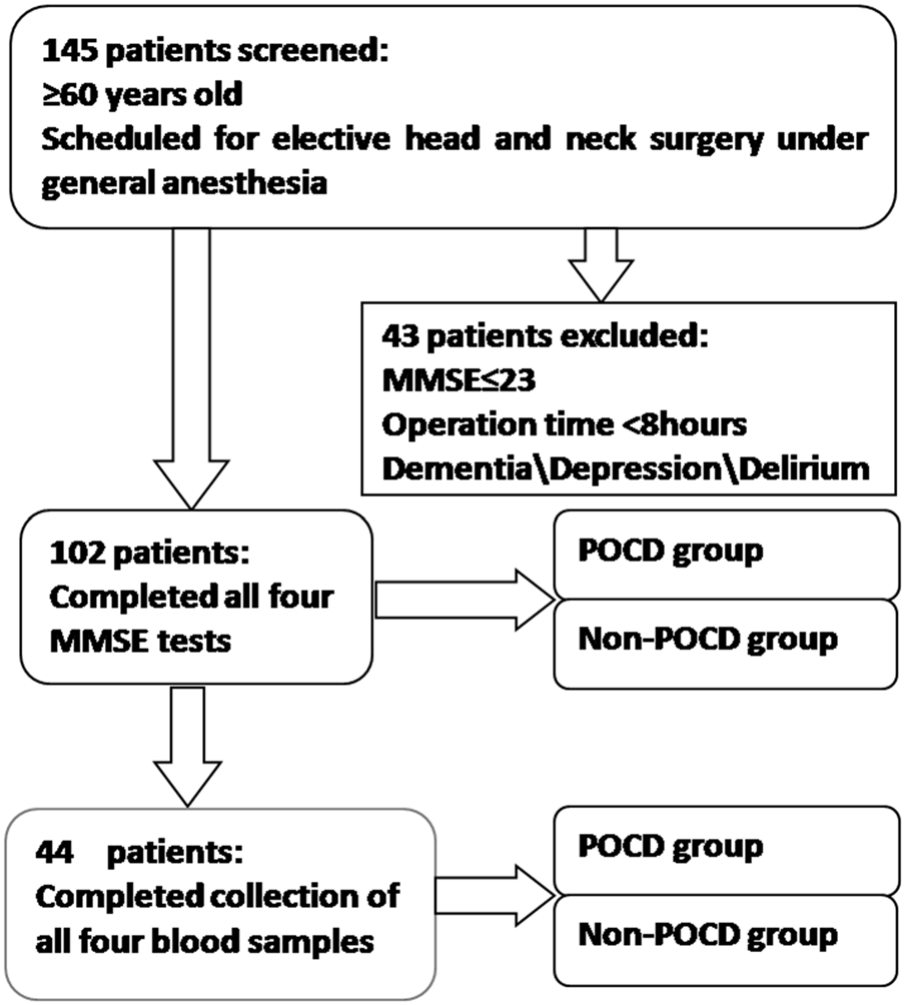
The trial flowchart

One hundred and forty-five patients were screened: ≥60 years old, scheduled for elective head and neck carcinoma surgery under general anesthesia. Forty-three patients were exclude for the operation time <8 h or preoperative MMSE score ≤23. A total 102 patients completed all four MMSE tests, Forty-four of 102 patients completed collection of all four blood samples, and were divided into two groups: POCD and non-POCD (Fig. 1).

There were no significant differences between the patients completing collection of all four blood samples and total patients in terms of age, sex, height, weight, BMI, education level, history of diabetes mellitus (DM), hypertension, smoking, drinking, cardiovascular disease, albumin, creatinine, blood sugar and operation time (*P* > 0.05). The incidence of POCD in patients undergoing elective head and neck carcinoma surgery under general anesthesia was 40.9 % (18/44) (Table 1).

**Table 1:** Characteristics of the total patients included in this study and patients completing collection of all four blood samples

	Total patients (n = 102)	Patients with blood collection (n = 44)	p
POCD	35	18	0.447
Gender (men %)	72 (70.6)	30 (68.2)	0.864
Age (y)	67.3 ± 5.9	67.4 ± 6.4	0.9636
Height (cm)	166.3 ± 6.8	166.7 ± 6.8	0.7523
Weight (kg)	64.6 ± 10.1	64.1 ± 11.0	0.7619
BMI (kg/m^2^)	23.4 ± 3.6	23.0 ± 3.5	0.5427
Education level			0.681
Primary school (%)	30 (29.4)	14 (31.8)	
Middle school (%)	56 (54.9)	21 (47.7)	
College or university (%)	16 (15.7)	9 (20.5)	
History of DM (%)	14 (13.7)	8 (18.2)	0.490
History of hypertension (%)	54 (52.9)	24 (54.5)	0.858
History of smoking (%)	19 (18.6)	10 (22.7)	0.569
History of drinking (%)	11 (10.8)	4 (9.1)	0.757
History of cardiovascular diseases (%)	29 (28.4)	12 (27.3)	0.886
Albumin (g/L)	41.0 ± 3.6	40.9 ± 4.4	0.8863
Creatinine (μmol/L)	88.0 ± 16.8	89.9 ± 13.7	0.5179
Blood sugar (mg/L)	5.1 ± 0.7	5.3 ± 0.7	0.1292
Operation time (h)	9.5 ± 1.4	9.9 ± 1.8	0.0851
MMSE^a^	27.9 ± 1.7	28.1 ± 1.9	0.472

Data are shown as mean ± SD or number ( %)

MMSE^a^: the score of MMSE on the day before operation

**Fig. 2:**
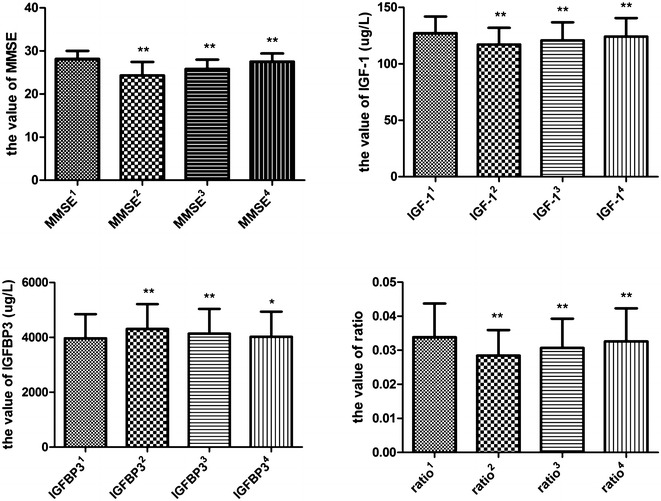
Perioperative changes of circulating IGF-1, IGFBP3, their ratio and MMSE scores. MMSE score on the day before operation (MMSE^1^), postoperative day 1(MMSE^2^), 3 (MMSE^3^) and 7(MMSE^4^); the circulating level of IGF-1 on the day before operation (IGF-1^1^), postoperative day 1 (IGF-1^2^), 3 (IGF-1^3^) and 7 (IGF-1^4^); the circulating level of IGFBP-3 on the day before operation (IGFBP3^1^), opostoperative day 1 (IGFBP3^2^), 3 (IGFBP3^3^) and 7 (IGFBP3^4^); and the ratio of IGF-1^1^ and IGFBP3^1^ (ratio^1^), the ratio of IGF-1^2^ and IGFBP3^2^ (ratio^2^), the ratio of IGF-1^3^ and IGFBP3^3^ (ratio^3^), the ratio of IGF-1^4^ and IGFBP3^4^ (ratio^4^). **P < 0.01, *P < 0.05 (compared with the value on the day before operation). *Bars* represent mean ± SD

Comparison of circulating IGF-1 level pre- and postoperatively showed that IGF-1^2^, IGF-1^3^ and IGF-1^4^ were significantly lower than IGF-1^1^ (117.13 ± 14.78, 120.78 ± 15.99, 124.15 ± 16.43 versus 127.20 ± 14.77 μg/L, *P* < 0.0001, *P* < 0.0001, *P* = 0.0006 respectively). The changes of MMSE scores and ratio of IGF-1/IGFBP3 were similar (24.32 ± 3.13, 25.82 ± 2.17, 27.50 ± 1.91 versus 28.14 ± 1.89, *P* < 0.0001, *P* < 0.0001, *P* = 0.0022 respectively; 0.0711 ± 0.0188, 0.0767 ± 0.0215, 0.08155 ± 0.02420 versus 0.0846 ± 0.0247, *P* < 0.0001, *P* < 0.0001, *P* = 0.0002 respectively). IGFBP3^2^, IGFBP3^3^ and IGFBP3^4^ were significantly higher than IGFBP3^1^ (4307.03 ± 904.16, 4139.42 ± 897.56, 4022.69 ± 913.42 versus 3968.82 ± 880.17 μg/L, *P* < 0.0001, *P* < 0.0001, *P* = 0.0325 respectively) (Fig. 2).

**Fig. 3:**
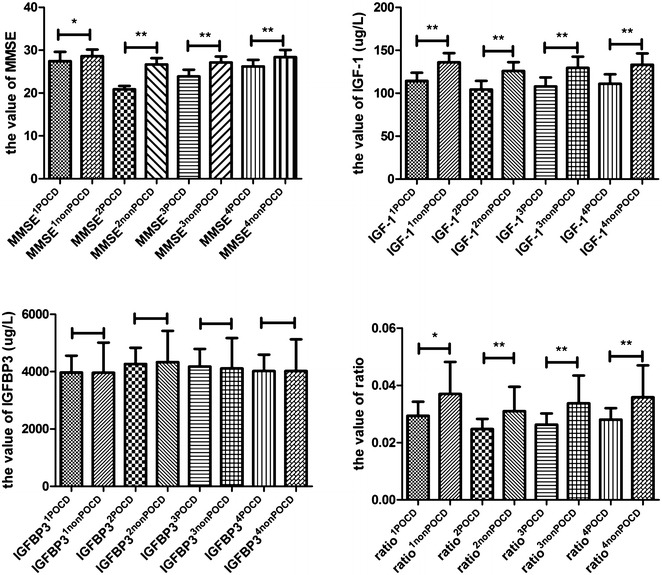
Circulating IGF-1, IGFBP3, their ratio and MMSE score in POCD and non-POCD group. MMSE score on the day before operation (MMSE^1^), postoperative day 1 (MMSE^2^), 3 (MMSE^3^) and 7 (MMSE^4^) in POCD and non-POCD groups; the circulating level of IGF-1 on the day before operation (IGF-1^1^), postoperative day 1 (IGF-1^2^), 3 (IGF-1^3^) and 7 (IGF-1^4^)day after operation in POCD and non-POCD groups; the circulating level of IGFBP3 on the day before operation (IGFBP3^1^), postoperative day 1 (IGFBP3^2^), 3 (IGFBP3^3^) and 7 (IGFBP3^4^) in POCD and non-POCD groups; and the ratio of IGF-1^1^ and IGFBP3^1^ (ratio^1^), the ratio of IGF-1^2^ and IGFBP3^2^ (ratio^2^), the ratio of IGF-1^3^ and IGFBP3^3^ (ratio^3^), the ratio of IGF-1^4^ and IGFBP3^4^ (ratio^4^) in POCD and non-POCD groups. **P < 0.01, *P < 0.05. *Bars* represent mean ± SD

Comparison of MMSE score, circulating IGF-1 and IGFBP3 levels and ratio of IGF-1/IGFBP3 between POCD group and non-POCD group showed that MMSE, IGF-1 and ratio were significant lower in the POCD group than non-POCD group, whereas there was no significant difference in IGFBP3^1^ (Fig. 3).

Among age, IGF-1^1^, IGFBP3^1^, ratio^1^ and MMSE^1^, there was a negative relationship between age and IGF-1^1^ (R = −0.3823, *P* = 0.0084), positive correlation between MMSE^1^ and IGF-1^1^ (R = 0.3743, *P* = 0.0123), positive correlation between MMSE^1^ and ratio^1^ (R = 0.3573, *P* = 0.0173), but IGFBP3^1^ was not significantly correlated with age (*R* = –0.2348, *P* = 0.1250) or MMSE^1^ (*R* = −0.1362, *P* = 0.3780), and MMSE^1^ and ratio^1^ was not significantly correlated with age (*R* = −0.08945, *P* = 0.3713, *R* = –0.07465, *P* = 0.6301 respectively).

Logistic regression analysis was performed to determine independent associations between particular parameters and the risk of POCD. Of the factors in the model, elderly patients, lower MMSE score, and preoperative circulating level of IGF-1 significantly increased the odds of POCD (OR = 1.39, *P* < 0.001; OR = 0.47, *P* = 0.004; OR = 0.87, *P* = 0.008 respectively).

## Discussion

Anesthetics can lead to cognitive impairment (Dwyer et al. 1992; Ghoneim and Block 1997). However, the mechanism of the influence of anesthetics on neurological function is not completely understood. In this study, the incidence of POCD was 40.9 % (18/44) on the 1st day after the operation, which is similar to the incidence in elderly patients undergoing orthopedic surgery (Gustafson et al. 1991).

Although IGF-1 is a multifunctional polypeptide essential for normal growth and development, IGF-I also plays an important role in neuroprotection. IGF-1 decreases the Aβ level in the brain (Carro et al. 2002) and induces inhibition of glycogen synthase kinase 3, which results in tau dephosphorylation and increased microtubule binding of tau (Hong and Lee 1997). In elderly humans, serum IGF-I levels positively correlate with cognitive status (Aleman et al. 1999), and a similar correlation in mice unveiled a trophic action of circulating IGF-I on glutamate neurotransmission affecting synaptic plasticity and cognition (Trejo et al. 2007). Namely, IGF-I improves learning and memory (Markowska et al. 1998). It has been demonstrated that exogenous IGF-1 both protects neurons from diverse forms of injury*in vivo* and *in vitro* (Cheng and Mattson 1992; Gluckman et al. 1998). IGF-1 can be produced in the brain (Alvarez et al. 2007), but it is mainly produced in the liver, and can enter the brain via the blood–brain barrier (Creyghton et al. 2004). According with previous report (Piriz et al. 2011), the present study revealed that circulating IGF-1 level negatively correlated with age, which gave evidence that circulating IGF-1 level decreased with increasing age. According with report of Aleman et al. (Aleman et al. 1999), change trends in circulating IGF-1 level were similar to those of MMSE score, and the circulating IGF-1 level positively correlated with MMSE score. At the same time, circulating IGF-1 level was significantly lower in POCD group than the level in non-POCD group. Hence, the down-regulation of circulating IGF-1 level may be involved in the mechanism of POCD. Furthermore, it was proved that cognitive function in Sprague–Dawley rats was reduced by sevoflurane accompanied by decreased expression of IGF-1 (Peng et al. 2011).

With regard to the association of IGF-I and IGFBP3 with cognition, Kalmijn et al. (Kalmijn et al. 2000) have disclosed that total IGF-I and total IGF-I/IGFBP3 molar ratio are negatively associated with cognitive decline. A further transverse study has also reported an association between free IGF-I, IGFBP3, and cognitive impairment among community-living elderly subjects, after adjusting for numerous potential confounders (age, sex, education, cerebrovascular disease, ischemic heart disease, congestive heart failure, hypertension, diabetes, depression, Parkinson’s disease, thyroid disease, smoking status, alcohol abuse, BMI, and number of medications) (Landi et al. 2007). In the present study, the circulating levels of IGF-1 (total IGF-1) and the ratio of IGF-1/IGBP3, indicative of bioavailability of IGF-1 significantly decreased and circulating IGFBP3 level significantly increased after surgery under general anesthesia, on the orther hand, IGF-1 levels and ratios were all significantly lower in POCD group than in non-POCD group at different times, whereas there was no significant difference in level of IGFBP3 between the patients with or without POCD. Further analysis showed that preoperative level of IGF-1 (total IGF-1) was independently, significantly associated with POCD, and lower preoperational circulating IGF-1 level (total IGF-1) increased the risk of POCD, but IGFBP3 and molar ratio were not significant determinants of POCD. Thus, it was the preoperative circulating level of IGF-1, and not IGFBP3 or their molar ratio, that negatively correlated with POCD in the present study, which suggested that preoperative circulating level independently affected the incidence of POCD. We showed that the relationship of IGF-1, IGFBP3, molar ratio of IGF-1/IGFBP3and POCD was not the same as their relationship with AD. The probable reason was that POCD was caused by the operation, anesthesia, and other relevant factors, whereas AD is a primary neurodegenerative disorder in the elderly population. There are some differences in their pathophysiological mechanism. Operation, anesthesia or other relevant factors down-regulated the circulating IGF-1 level [maybe inhibited the synthesis of IGF-1 in liver based on the previous report that it is mainly produced in the liver, and can enter the brain via the blood–brain barrier (Creyghton et al. 2004)], then decreased the amount of IGF-1 entering to brain through the blood–brain barrier. Decrease of IGF-1 in brain leaded to attenuate ILPs function, increase of Aβ level in the brain and tau hyperphosphorylation and decrease of microtubule binding of tau, ultimately impaired cognitive function. However, this hypothesis is needed to be proved in the future researches.

In conclusion, the present study provides evidence that postoperative circulating IGF-1 level (total IGF-1) and the ratio of IGF-1/IGFBP3 (bioavalable IGF-1) were significantly lower than preoperative level, whereas postoperative circulating IGFBP3 level was higher. Elderly patients have lower circulating IGF-1 levels and are more susceptible to POCD. Lower preoperative MMSE score and the circulating level of IGF-1, not the ratio or IGFBP3 level, significantly increase the risk of POCD. Down-regulation of circulating IGF-1 level may be involved in the mechanism of POCD. Preoperative measurement of MMSE and circulating level of IGF-1 are likely to be useful in screening for onset of POCD.
